# Maternal Nicotine Exposure Leads to Impaired Disulfide Bond Formation and Augmented Endoplasmic Reticulum Stress in the Rat Placenta

**DOI:** 10.1371/journal.pone.0122295

**Published:** 2015-03-26

**Authors:** Michael K. Wong, Catherine J. Nicholson, Alison C. Holloway, Daniel B. Hardy

**Affiliations:** 1 Department of Physiology and Pharmacology, Western University, London, Ontario, Canada; 2 Department of Obstetrics and Gynecology, McMaster University, Hamilton, Ontario, Canada; 3 Departments of Obstetrics and Gynecology, Children’s Health Research Institute, Lawson, Health Research Institute, Western University, London, Ontario, Canada; University of Hong Kong, HONG KONG

## Abstract

Maternal nicotine exposure has been associated with many adverse fetal and placental outcomes. Although underlying mechanisms remain elusive, recent studies have identified that augmented endoplasmic reticulum (ER) stress is linked to placental insufficiency. Moreover, ER function depends on proper disulfide bond formation—a partially oxygen-dependent process mediated by protein disulfide isomerase (PDI) and ER oxidoreductases. Given that nicotine compromised placental development in the rat, and placental insufficiency has been associated with poor disulfide bond formation and ER stress, we hypothesized that maternal nicotine exposure leads to both placental ER stress and impaired disulfide bond formation. To test this hypothesis, female Wistar rats received daily subcutaneous injections of either saline (vehicle) or nicotine bitartrate (1 mg/kg) for 14 days prior to mating and during pregnancy. Placentas were harvested on embryonic day 15 for analysis. Protein and mRNA expression of markers involved in ER stress (*e*.*g*., phosphorylated eIF2α, Grp78, Atf4, and CHOP), disulfide bond formation (*e*.*g*., PDI, QSOX1, VKORC1), hypoxia (Hif1α), and amino acid deprivation (GCN2) were quantified via Western blot and/or Real-time PCR. Maternal nicotine exposure led to increased expression of Grp78, phosphorylated eIF2α, Atf4, and CHOP (p<0.05) in the rat placenta, demonstrating the presence of augmented ER stress. Decreased expression of PDI and QSOX1 (p<0.05) reveal an impaired disulfide bond formation pathway, which may underlie nicotine-induced ER stress. Finally, elevated expression of Hif1α and GCN2 (p<0.05) indicate hypoxia and amino acid deprivation in nicotine-exposed placentas, respectively, which may also cause impaired disulfide bond formation and augmented ER stress. This study is the first to link maternal nicotine exposure with both placental ER stress and disulfide bond impairment *in vivo*, providing novel insight into the mechanisms underlying nicotine exposure during pregnancy on placental health.

## Introduction

Despite increased awareness and education, approximately 10–28% of women were reported to continue smoking during pregnancy [1–4]. Cigarette smoke contains many teratogens, and exposure during pregnancy increases the risk of adverse pregnancy and neonatal outcomes, including placental abruption, placenta previa, sudden infant death syndrome, spontaneous abortion, stillbirth, low birth weight, and fetal growth restriction [5–11]. Although many pregnant women want to quit smoking, recent studies suggest that only 50% successfully abstain from smoking during pregnancy due in part to the highly addictive nature of nicotine [12, 13].

Nicotine replacement therapies (*e*.*g*., nicotine patches and gums) were developed to assist with smoking cessation while concurrently allowing the smoker to avoid the thousands of chemicals in cigarette smoke [14]. Although nicotine replacement therapies are often considered to be safer than smoking, there is still great concern regarding the effects of nicotine on fetal and postpartum health (please refer to [15] for a review).

Due to the lipid-soluble nature of nicotine, it easily traverses membrane barriers to enter the placenta, where it can bind to many subtypes of nicotinic acetylcholine receptors (nAChR) previously reported to be expressed in human and rat placental syncytiotrophoblasts, cytotrophoblasts, Hofbauer cells, visceral yolk sac epithelium, and amniotic epithelium [16,17]. Emerging animal studies demonstrate that nicotine *alone* during pregnancy can lead to compromised placental and fetal development, with many detrimental health outcomes in the offspring [18–24]. Furthermore, nicotine exposure *in utero* results in low birth weight pups, implicating placental insufficiency [25–27]. Specifically, nicotine *in utero* led to compromised placental development in pregnant rat dams (*e*.*g*., compacted decidual and junctional zones, decreased labyrinth vascularization and cell proliferation, increased placental hypoxia, and impaired trophoblast differentiation) at embryonic day 15, prior to any observable fetal growth deficiencies [17]. Given that compromised placental development is a strong predictor of fetal growth restriction in humans, elucidation of the underlying mechanisms would be therapeutically beneficial for offspring exposed to nicotine *in utero* [28]. However, to date, the mechanisms linking maternal nicotine exposure to compromised placental function remain elusive.

Recent studies have suggested that endoplasmic reticulum (ER) stress, the perturbation of ER homeostasis due to the accumulation of misfolded or unfolded proteins, plays a critical role underlying compromised placentation [29–33]. The unfolded protein response (UPR) activates to alleviate the stress and restore ER homeostasis through three major signalling pathways governed by activating transcription factor 6 (Atf6), inositol-requiring enzyme 1α endoribonuclease (IRE1α), and protein kinase-like endoplasmic reticulum kinase (PERK) [34–36]. However, if the ER remains severely debilitated, C/EBP-homologous protein/Gadd153 (CHOP) activates downstream apoptosis [37, 38], which has been associated with compromised placental growth both *in vivo* and *in vitro* [31, 39]. Since the ER is the primary site of protein synthesis and maturation within the cell, prolonged disturbance of its function through ER stress could negatively impact essential signalling and transport function in the placenta (*e*.*g*., VEGF and Glut-1 expression) [29, 40–43]. Moreover, one of the key processes underlying protein maturation and folding within the ER lumen is the disulfide bond formation of nascent proteins through protein thiol oxidation, and deterrence of its function has been demonstrated to augment ER stress [40, 44–46]. It is important to note that due to high protein secretory activity in the placenta, low-moderate basal levels of UPR activation may occur even under normal physiological conditions; however, chronic pathological augmentation may rear consequences in placental development [29, 33, 47].

Nicotine is known to induce vasoconstriction in placental and umbilical vasculature, thus restricting oxygen and nutrient (*e*.*g*., amino acids) supply [48, 49]. Interestingly, hypoxia and low amino acid supply have been demonstrated to both hinder disulfide bond formation and induce ER stress *in vitro* [50–54]. Exposure to cigarette smoke or nicotine has also been shown to cause ER stress in several tissue/cell types, but little is known about the mechanisms underlying maternal nicotine exposure *in vivo* on ER stress and disulfide bond formation in the developing placenta [55–61]. Therefore, the aim of this study was to determine whether maternal nicotine exposure *in vivo* leads to augmented placental ER stress and impaired disulfide bond formation in the rat placenta.

## Materials and Methods

### Experimental model

All animal experiments were approved by the Animal Research Ethics Board at McMaster University in accordance with the guidelines of the Canadian Council for Animal Care. Nulliparous female Wistar rats (200–250 g, Harlan, Indianapolis, IN, USA) were randomly assigned to receive daily subcutaneous injections of either saline (vehicle) (n = 6) or nicotine bitartrate (1 mg/kg, Sigma-Aldrich) (n = 5) for 14 days prior to mating and during pregnancy. This dose has previously resulted in maternal and neonatal serum cotinine concentrations similar to either moderate female smokers and/or low-dose nicotine replacement therapy users [24, 62–64]. Mating (embryonic day (e) 0) was confirmed by the presence of sperm in a vaginal flush. At necropsy (e15) whole placentas were harvested, immediately flash-frozen in liquid nitrogen, and stored at -80°C until molecular analyses were performed.

### RNA extraction and Real Time-Polymerase Chain Reaction (RT-PCR)

Total RNA was extracted from homogenized whole placentas using TRIzol reagent (Invitrogen). Chloroform (Sigma-Aldrich) was added to the solution, and then centrifuged at 12,500rpm. Supernatant was transferred to a fresh tube with an equal volume of isopropanol (Sigma-Aldrich) and centrifuged again at 12,500rpm. Total RNA was then collected from the pellet and dissolved in DEPC-treated water. Deoxyribonuclease I, Amplification Grade (Invitrogen) was added to the RNA to digest contaminating single- and double-stranded DNA. Four μg of RNA were reverse-transcribed to cDNA using random hexamers and Superscript II Reverse Transcriptase (Invitrogen). Primer sets directed against gene targets of interest were designed through National Center for Biotechnology Information’s primer designing tool and generated via Invitrogen Custom DNA Oligos (Table 1). Quantitative analysis of mRNA expression was performed via RT-PCR using fluorescent nucleic acid dye SsoFast EvaGreen supermix (BioRad) and BioRad CFX384 Real Time System. The cycling conditions were 95°C for 10 min, followed by 43 cycles of 95°C for 15 sec and 60°C for 30 sec and 72°C for 30 sec. The cycle threshold was set so that exponential increases in amplification were approximately level between all samples. Relative fold changes were calculated using the comparative cycle times (Ct) method, normalizing all values to the geometric means of three housekeeping genes (β-Actin, 18S, and Gapdh). Suitable housekeeping genes were determined using algorithms from GeNorm [65], Normfinder [66], BestKeeper [67], and the comparative ΔCt method [68] to provide an overall ranking of the most stable housekeeping genes (available online at http://www.leonxie.com/referencegene.php) (Please refer to S1 Fig. to see all mRNA targets normalized to individual housekeeping genes). Given all primer sets had equal priming efficiency, the ΔCt values for each primer set were calibrated to the experimental samples with the lowest transcript abundance (highest Ct value), and the relative abundance of each primer set compared with calibrator was determined by the formula 2^ΔΔCt^, in which ΔΔCt was the normalized value.

**Table 1:** Forward and reverse sequences for the primers used for quantitative Real-Time PCR.

*Gene*	*Forward*	*Reverse*	*GenBank/Reference*
Atf6	GGATTTGATGCCTTGGGAGTCAGAC	ATTTTTTTCTTTGGAGTCAGTCCAT	NM_001107196.1
Xbp1	GAGCAGCAAGTGGTGGAT	TCTCAATCACAAGCCCATG	NM_001004210.2
Spliced Xbp1	GAGTCCGCAGCAGGTG	GCGTCAGAATCCATGGGA	(69)
Grp78	AACCCAGATGAGGCTGTAGCA	ACATCAAGCAGAACCAGGTCAC	NM_013083.2
Atf4	CCTGACTCTGCTGCTTATATTACTCTAAC	ACTCCAGGTGGGTCATAAGGTTTG	NM_024403.2
CHOP	CCAGCAGAGGTCACAAGCAC	CGCACTGACCACTCTGTTTC	NM_001109986.1
PRDX4	TCCTGTTACAGACTGAAGCTTTGC	GTGATCTGCGACCGAAACCC	NM_053512.2
GPx-7	CCTGCCTTCAAATACCTAACCC	TGTAATACGGGGCTTGATCTCC	NM_001106673.1
VKORC1	GCTGGTGGAGCATGTGTTAGG	CAACGTCCCCTCAAGCAACC	NM_203335.2
QSOX1	AGCCACTGCCCTAGATGTACC	TGAGGCCTGCGTTTAGTTCC	NM_001109898.1
Bax	AGGATCGAGCAGAGAGGATGG	GACACTCGCTCAGCTTCTTGG	NM_017059.2
Bcl-2	TGTGGATGACTGAGTACCTGAACC	CAGCCAGGAGAAATCAAACAGAGG	NM_016993.1
β-Actin	CACAGCTGAGAGGGAAAT	TCAGCAATGCCTGGGTAC	NM_031144
18S	TTGCTGATCCACATCTGCTGG	ATTGCCGACAGGATGCAGAA	M11188.1
Gapdh	GGATACTGAGAGCAAGAGAGAGG	TCCTGTTGTTATGGGGTCTGG	NM_017008.4

### Xbp1 splicing assay

Quantitative analysis of Xbp1 mRNA splicing was performed as previously described [69]. Briefly, primers were designed to span the unique exon-exon border formed by unconventional IRE1 splicing to target spliced Xbp1 mRNA. Primers were also designed to target total Xbp1 mRNA (Table 1). Results were normalized to the geometric means of three housekeeping genes (β-Actin, 18S, and Gapdh), and then a ratio of spliced to total Xbp1 was taken to quantify the splicing of Xbp1.

### Protein extraction and Western blot

Whole placentas were homogenized in RIPA buffer (50 mM Tris-HCL, pH 7.4, 150 mM NaCl, 1 mM EDTA, 1% Nonidet P40, 0.25% C_24_H_39_NaO_4_, supplemented with phosphatase inhibitors (20 mM NaF, 40mM Na-pyrophosphate, 40mM Na_3_VO_4_, 200mM β-glycerophosphate disodium salt hydrate), and a protease inhibitor cocktail (Roche)). The solution was sonicated at 30% amplitude for 5 sec total, 1 sec per pulse. It was then mixed in a rotator for 10 min at 4°C and centrifuged at 300g for 15 min at 4°C. The supernatant was collected and centrifuged at 16000g for 20 min at 4°C. The resulting supernatant was collected as the total cellular protein extract and quantified by colorimetric DC protein assay (BioRad). Loading samples were prepared with fresh total cellular protein extract (avoiding repeated freeze-thaw cycles), NuPAGE LDS Sample Buffer (4X) (Invitrogen), NuPAGE Reducing Agent (10X) (Invitrogen), and deionized water, and heated at 70°C for 10 min to denature the proteins. Proteins (20μg/well) were separated by size via gel electrophoresis in gradient polyacrylamide gels (Novex), and transferred onto polyvinylidene difluoride membrane (Millipore). Membranes were blocked in 1x Tris-buffered saline-Tween 20 buffer with 5% non-fat milk (blocking solution), and then probed using primary antibodies of the protein targets of interest, all diluted in the blocking solution (Table 2). Secondary antibodies were used to detect the species-specific portion of the primary antibody, all diluted in the blocking solution (Table 3). Immuno-reactive bands were visualized using SuperSignal West Dura Chemiluminescent Substrate (Thermo Scientific).

**Table 2:** Western Blot primary antibodies, dilutions used in experiments, and company and catalogue information.

*Antibody name*	*Source*	*Dilution*	*Company (#Catalogue)*
KDEL (Grp78) (10C3)	Mouse monoclonal	1:300	Santa Cruz Biotechnology Inc., Santa Cruz, CA, USA (#sc-58774)
Atf6	Mouse monoclonal	1:600	Novus Biologicals, Oakville, ON, Canada (NBP1-40256)
Phospho-PERK (Thr980) (16F8)	Rabbit monoclonal	1:500	Cell Signaling Technology Inc., Danvers, MA, USA (#3179)
PERK (D11A8)	Rabbit monoclonal	1:500	Cell Signaling Technology Inc., Danvers, MA, USA (#5683)
Phospho-eIF2α (Ser51) (119A11)	Rabbit monoclonal	1:1000	Cell Signaling Technology Inc., Danvers, MA, USA (#3597)
eIF2α	Rabbit monoclonal	1:1000	Cell Signaling Technology Inc., Danvers, MA, USA (#9722)
CREB-2 (Atf4) (C-20)	Rabbit polyclonal	1:5000	Santa Cruz Biotechnology Inc., Santa Cruz, CA, USA (#sc-200)
CHOP (D46F1)	Rabbit monoclonal	1:500	Cell Signaling Technology Inc., Danvers, MA, USA (#5554)
Ero1-Lα	Rabbit polyclonal	1:1000	Cell Signaling Technology Inc., Danvers, MA, USA (#3264)
PDI (C81H6)	Rabbit monoclonal	1:1000	Cell Signaling Technology Inc., Danvers, MA, USA (#3501)
VKORC1 (D-17)	Rabbit polyclonal	1:500	Santa Cruz Biotechnology Inc., Santa Cruz, CA, USA (#sc-54456-R)
Quiescin Q6 (QSOX1) (G-12)	Goat polyclonal	1:500	Santa Cruz Biotechnology Inc., Santa Cruz, CA, USA (#sc-160084)
GPx-7 (S-12)	Goat polyclonal	1:500	Santa Cruz Biotechnology Inc., Santa Cruz, CA, USA (#sc-160062)
Caspase-3 (8G10)	Rabbit monoclonal	1:1000	Cell Signaling Technology Inc., Danvers, MA, USA (#9665)
Caspase-6	Rabbit polyclonal	1:1000	Cell Signaling Technology Inc., Danvers, MA, USA (#9762)
Caspase-7 (D2Q3L)	Rabbit monoclonal	1:1000	Cell Signaling Technology Inc., Danvers, MA, USA (#12827)
Lamin A/C (4C11)	Mouse monoclonal	1:1000	Cell Signaling Technology Inc., Danvers, MA, USA (#4777)
Bax	Rabbit polyclonal	1:500	Santa Cruz Biotechnology Inc., Santa Cruz, CA, USA (#sc-493)
Bcl-2	Rabbit polyclonal	1:100	Abcam Inc., Toronto, ON, Canada (#ab7973)
β-Actin	Mouse monoclonal	1:50000	Sigma-Aldrich Co., St. Louis, MO, USA Canada (#A3854)

**Table 3:** Western Blot secondary antibodies, dilutions used in experiments, and company and catalogue information.

*Antibody name*	*Dilution*	*Company (#Catalogue)*
Donkey Anti-Rabbit IgG (H+L)	1:10000	Jackson ImmunoResearch Laboratories, West Grove, PA, USA (#711-001-003)
Donkey Anti-Mouse IgG (H+L)	1:5000	Jackson ImmunoResearch Laboratories, West Grove, PA, USA (#715-001-003)
Donkey Anti-Goat IgG (H+L)	1:5000	Jackson ImmunoResearch Laboratories, West Grove, PA, USA (#705-001-003)

### Statistical analysis

All statistical analyses were performed using GraphPad Prism 5 software. All results were expressed as means of normalized values ± SEM. Significant outliers were statistically identified using the Grubbs’ test [70]. The significance of the differences (p<0.05) between normalized mean values was then evaluated using the two-tailed, nonparametric Mann-Whitney test.

## Results

### Maternal nicotine exposure leads to augmented ER stress and unfolded protein response activation in embryonic day 15 placenta

**Fig 1:**
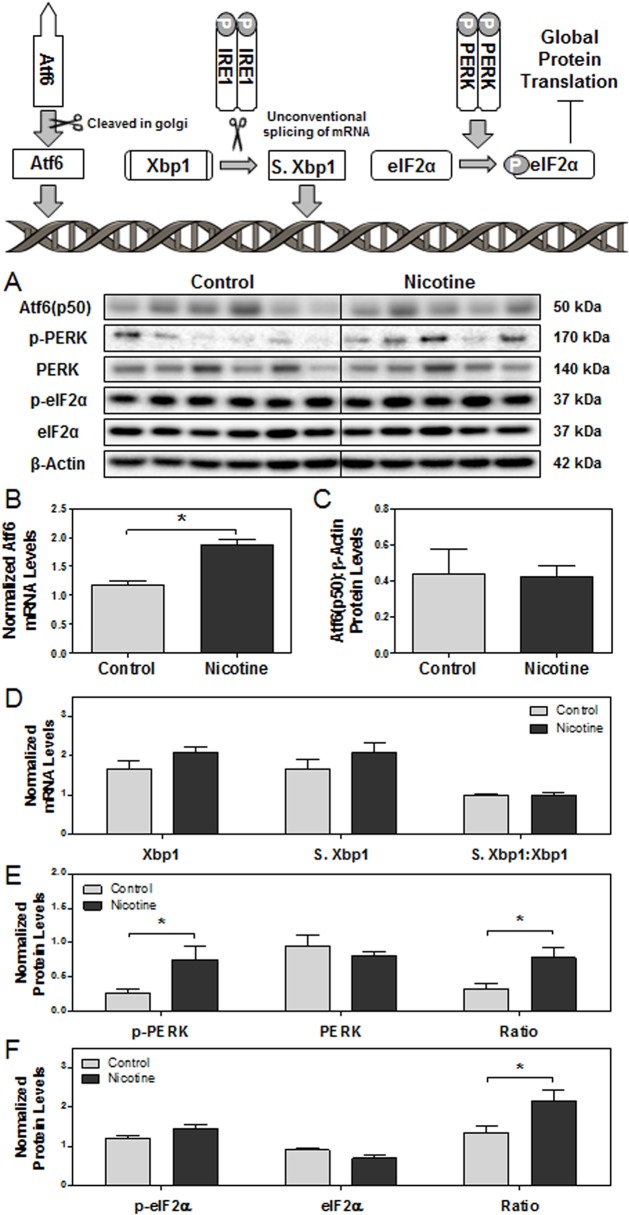
The effect of maternal nicotine exposure on the three branches of the unfolded protein response (Atf6, IRE1α, PERK) in e15 rat placentas. Protein and mRNA levels of targets of interest were determined via Western blot and RT-PCR, respectively. **(A)** Specific targeted protein bands as detected by respective antibodies via Western blot. **(B)** Atf6 mRNA levels. **(C)** Atf6 protein levels. **(D)** mRNA levels of spliced Xbp1, unspliced Xbp1, and ratio of spliced: unspliced Xbp1. **(E)** Protein levels of p-PERK [Thr980], PERK, and ratio of p-PERK: PERK. **(F)** Protein levels of p-eIF2α [Ser51], eIF2α, and ratio of p-eIF2α:eIF2α. All protein levels were expressed as means normalized to β-Actin ± SEM (n = 5-6/group). All mRNA levels were expressed as means normalized to the geometric mean of three stable housekeeping genes (β-Actin, 18S, and Gapdh) ± SEM (n = 5-6/group). *, Significant difference (p < 0.05). **, Significant difference (p<0.01).

To determine the presence of ER stress in nicotine-exposed placenta, we assessed mRNA and protein levels of the main players involved in the three branches of the UPR (Atf6, IRE1α, and PERK) via Real-Time PCR and Western blot, respectively. Activation of the UPR indicates the presence of ER stress [35]. With respect to the Atf6 branch of the UPR, the steady-state mRNA levels of Atf6 were found to be significantly elevated in e15 nicotine-treated placentas compared to controls (p<0.05), however, the protein levels of active Atf6(p50) remained unaltered (Fig. 1A-C). To determine the activation of the IRE1α branch, splicing of its downstream target, Xbp1 mRNA, was measured and found to be unaltered (Fig. 1D). However, nicotine exposure led to activation of the PERK branch of the UPR as demonstrated through significantly increased ratios of phosphorylated PERK [Thr980]: total PERK protein levels in nicotine-exposed placentas compared to controls at e15 (p<0.05, Fig. 1A, E). Nicotine exposure also led to significantly increased ratios of phosphorylated eukaryotic initiation factor (eIF) 2α [Ser51]: total eIF2α protein levels in the placenta (p<0.05), suggesting global protein translation attenuation (Fig. 1A, F).

**Fig 2:**
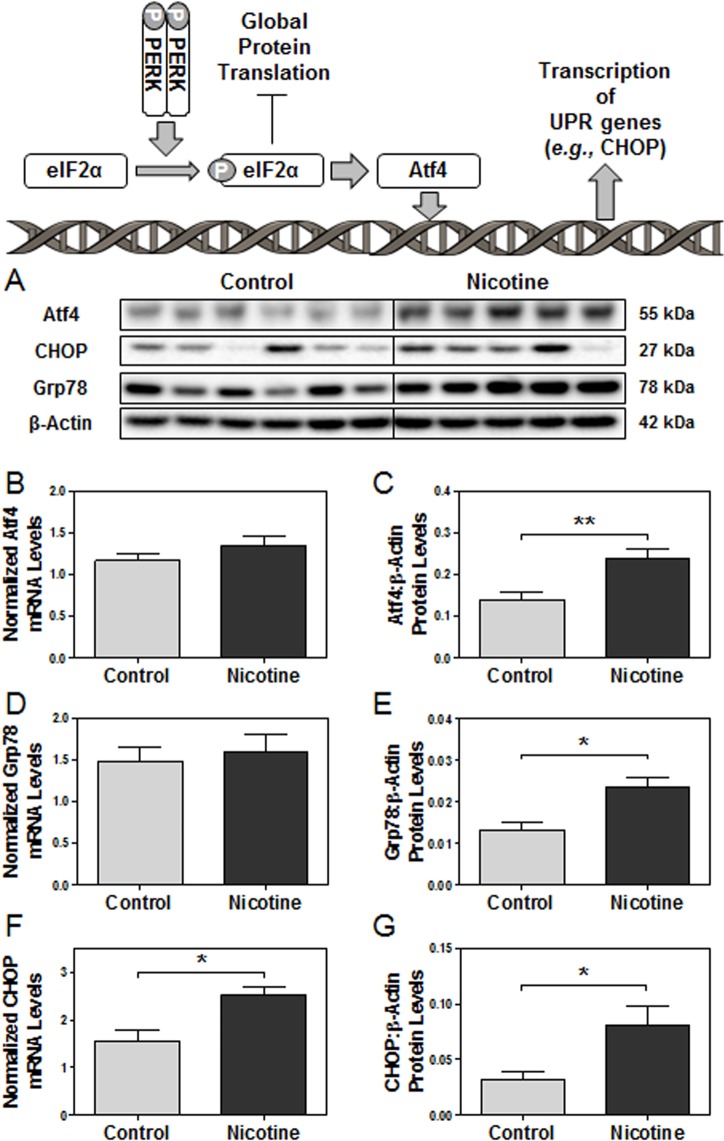
Nicotine exposure leads to activation of downstream targets in the PERK branch of the unfolded protein response in e15 rat placentas. Protein and mRNA levels of targets of interest were determined via Western blot and RT-PCR, respectively. **(A)** Specific targeted protein bands as detected by respective antibodies via Western blot. **(B)** Atf4 mRNA levels. **(C)** Atf4 protein levels. **(D)** Grp78 mRNA levels. **(E)** Grp78 protein levels. **(F)** CHOP mRNA levels. **(G)** CHOP protein levels. All protein levels were expressed as means normalized to β-Actin ± SEM (n = 5-6/group). All mRNA levels were expressed as means normalized to the geometric mean of three stable housekeeping genes (β-Actin, 18S, and Gapdh) ± SEM (n = 5-6/group).*, Significant difference (p < 0.05). **, Significant difference (p<0.01).

Since activation of the PERK pathway of the unfolded protein response was demonstrated, we decided to next investigate the expression of its potential downstream targets, Atf4, Grp78, and CHOP. Atf4 protein levels were significantly elevated in e15 nicotine-exposed placentas compared to controls (p<0.01) with unchanged steady-state mRNA levels (Fig. 2A-C). Grp78 protein levels were also significantly elevated in nicotine-exposed placentas (p<0.05) with unchanged mRNA levels (Fig. 2A, D-E), revealing post-transcriptional ER-stress-related increases in protein expression. However, nicotine exposure led to increased expression of CHOP (p<0.05), indicating prolonged ER stress and potential activation of ER-stress-related apoptotic pathways in e15 placentas (Fig. 2A, F-G). Collectively, these results confirm the presence of augmented ER stress and unfolded protein response activation of the PERK pathway in nicotine-treated placentas.

### The effects of nicotine-induced activation of CHOP on downstream apoptotic pathways

**Fig 3:**
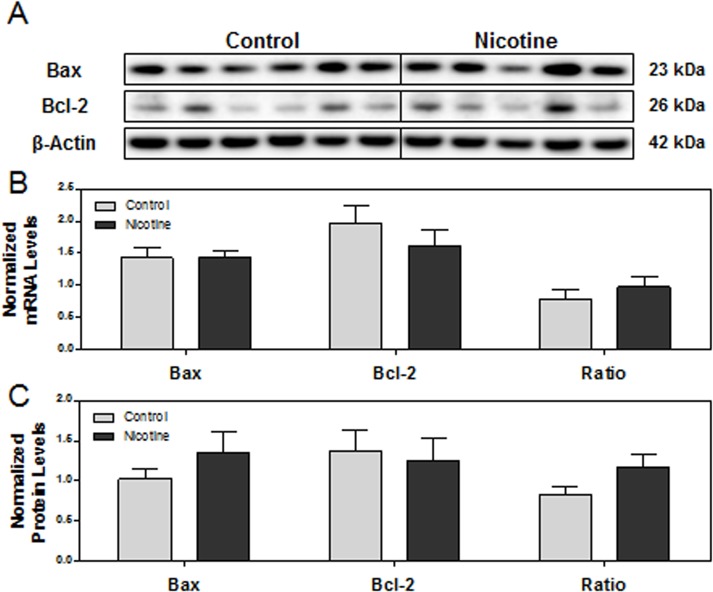
The effect of maternal nicotine exposure on downstream CHOP-mediated apoptotic pathways. Protein and mRNA levels of targets of interest were determined via Western blot and RT-PCR, respectively. **(A)** Specific targeted protein bands as detected by respective antibodies via Western blot. **(B)** mRNA levels of Bax, Bcl-2, and ratio of Bax: Bcl-2. **(C)** Protein levels of Bax, Bcl-2, and ratio of Bax: Bcl-2. All protein levels were expressed as means normalized to β-Actin ± SEM (n = 5-6/group). All mRNA levels were expressed as means normalized to the geometric mean of three stable housekeeping genes (β-Actin, 18S, and Gapdh) ± SEM (n = 5-6/group).

Due to elevated expression of CHOP in nicotine-exposed placentas, we next wanted to determine the expression of downstream apoptotic targets, pro-apoptotic Bax and anti-apoptotic Bcl-2, which are known to synergistically orchestrate apoptosis [37, 71]. To assess the level of apoptotic activation, the ratio of Bax: Bcl-2 mRNA levels were quantified; however, we found no significant difference between groups (Fig. 3A, B). There was a slight increase in Bax protein and decrease in Bcl-2 protein in nicotine-exposed placentas compared to controls, however, neither the markers nor their ratio reached statistically significant differences (Fig. 3A, C).

### Maternal nicotine exposure does not induce caspase-mediated apoptosis

**Fig 4:**
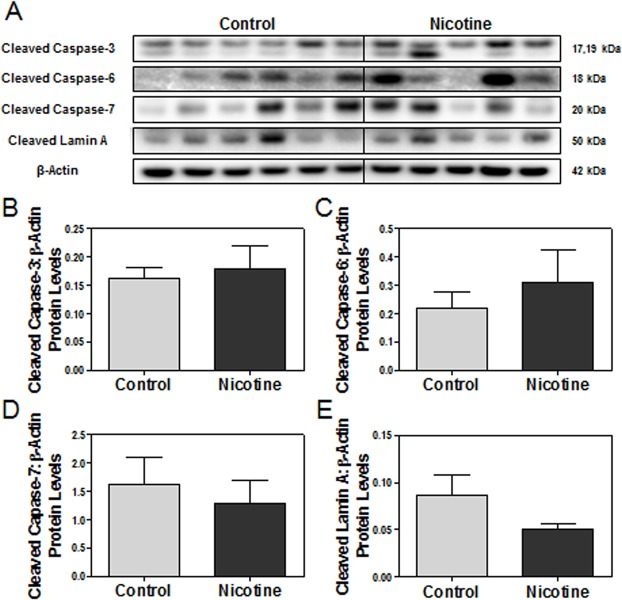
The effect of maternal nicotine exposure on downstream caspase-mediated apoptotic pathways. Protein levels of targets of interest were determined via Western blot. **(A)** Specific targeted protein bands as detected by respective antibodies via Western blot. **(B)** Cleaved caspase-3 protein levels. **(C)** Cleaved caspase-6 protein levels. **(D)** Cleaved caspase-7 protein levels. **(E)** Cleaved Lamin A protein levels. All protein levels were expressed as means normalized to β-Actin ± SEM (n = 5-6/group).

To further investigate the severity of the ER stress, we measured another major ER stress-related apoptotic pathway, the caspase-mediated apoptosis pathway. We found no significant differences in the protein levels of cleaved caspase-3, 6, 7, nor their substrate Lamin A, between control and nicotine-exposed placentas at e15 (Fig. 4).

### Maternal nicotine exposure down-regulates expression of protein disulfide isomerase and ER oxidoreductases

**Fig 5:**
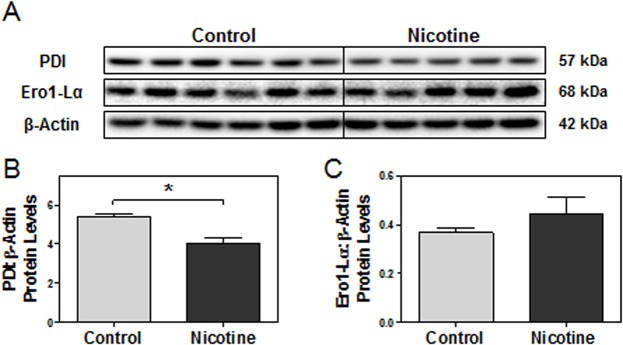
Nicotine decreases PDI expression in e15 rat placentas. Protein levels of targets of interest were determined via Western blot. **(A)** Specific targeted protein bands as detected by respective antibodies via Western blot. **(B)** PDI protein levels. **(C)** Ero1-Lα protein levels. All protein levels were expressed as means normalized to β-Actin ± SEM (n = 5-6/group). *, Significant difference (p<0.05).

In order to elucidate the underlying mechanisms of nicotine-induced ER stress in the placenta, we further examined disulfide bond formation, a process intimately connected with ER homeostasis and known to cause ER stress when compromised [52]. Specifically, we were interested in looking at the effects of nicotine on expression of the key isomerase and oxidoreductases that carry out disulfide bond formation. Protein disulfide isomerase (PDI) mediates protein folding by introducing disulfide bonds to nascent proteins through thiol oxidation. ER oxidoreductase, Ero1-Lα/β, will then subsequently reoxidize PDI to continue the redox relay [40, 44, 45]. Interestingly, PDI protein levels were found to be significantly decreased in nicotine-exposed placentas at e15 (p<0.05, Fig. 5A, B). In contrast, the main ER oxidoreductase, Ero1-Lα, demonstrated no significant change between treatment groups (Fig. 5A, C).

**Fig 6:**
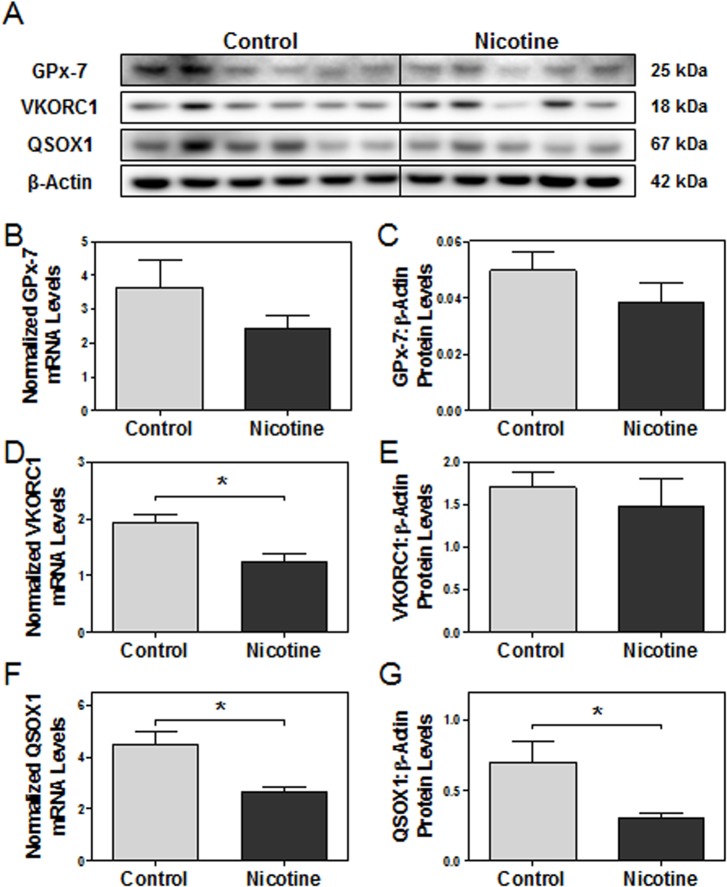
The effect of maternal nicotine exposure on various ER oxidoreductases in e15 rat placentas. Protein and mRNA levels of targets of interest were determined via Western blot and RT-PCR, respectively. **(A)** Specific targeted protein bands as detected by respective antibodies via Western blot. **(B)** GPx-7 mRNA levels. **(C)** GPx-7 protein levels. **(D)** VKORC1 mRNA levels. **(E)** VKORC1 protein levels. **(F)** QSOX1 mRNA levels. **(G)** QSOX1 protein levels. All protein levels were expressed as means normalized to β-Actin ± SEM (n = 5-6/group). All mRNA levels were expressed as means normalized to the geometric mean of three stable housekeeping genes (β-Actin, 18S, and Gapdh) ± SEM (n = 5-6/group). *, Significant difference (p<0.05).

These results provoked further exploration of the expression of alternative ER oxidoreductases (*e*.*g*., PRDX4, GPx-7, VKORC1, and QSOX1) recently found to be involved in PDI reoxidation and/or direct thiol oxidation of nascent proteins [72–77]. Real-time PCR revealed decreases in the steady-state mRNA levels of GPx-7, VKORC1 (p<0.05) and QSOX1 (p<0.05, Fig. 6A, B, D, F). Additionally, QSOX1 was significantly decreased at the protein level in nicotine-treated placentas compared to the controls (p<0.05, Fig. 6A, G).

### Maternal nicotine exposure increases markers of hypoxia and amino acid deprivation

**Fig 7:**
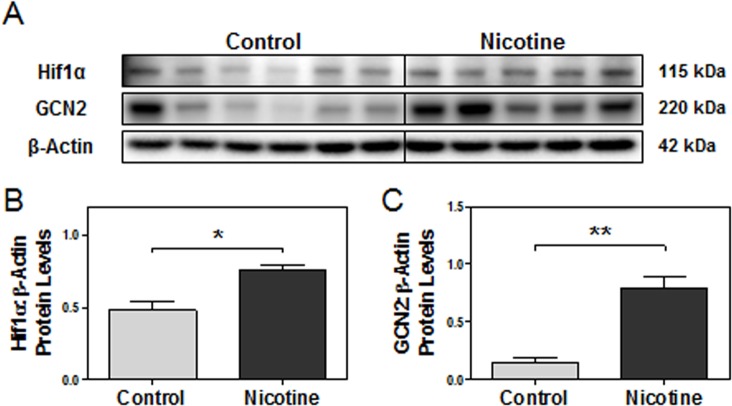
Nicotine-induced vasoconstriction leads to both hypoxia and reduced amino acid supply in e15 placenta. Protein levels of targets of interest were determined via Western blot. **(A)** Specific targeted protein bands as detected by respective antibodies via Western blot. **(B)** Hif1α protein levels. **(C)** GCN2 protein levels. All protein levels were expressed as means normalized to β-Actin ± SEM (n = 5-6/group). *, Significant difference (p<0.05). **, Significant difference (p<0.01).

Given that oxygen is the final electron acceptor in post-translational disulfide bond formation [52], we next investigated whether hypoxia was induced by nicotine exposure by measuring placental protein levels of hypoxia-inducible factor (Hif) 1α [78, 79]. Western blot revealed that Hif1α protein levels were significantly elevated in e15 nicotine-treated placentas compared to the controls (p<0.05, Fig. 7A, B). We were also interested in exploring whether additional insults induced by the vasoconstrictive effects of nicotine were present (*e*.*g*., low amino acid supply) [48]. General control non-depressible 2 (GCN2) is a protein kinase that responds to amino acid starvation by up-regulating transcription factors (*e*.*g*., GCN4) to mediate the nutrient deprivation. GCN2 also acts as an alternative kinase to phosphorylate eIF2α alongside PERK to attenuate protein translation [80, 81]. Western blot revealed that GCN2 protein levels were strongly elevated in e15 nicotine-treated placentas compared to the controls (p<0.01, Fig. 7A, C).

## Discussion

In the current study, we have demonstrated that nicotine exposure in pregnant rats leads to augmented ER stress in the e15 placenta. We were interested in selecting a time-point during pregnancy when nicotine exposure was previously shown to cause structural and morphological aberrations in the rat placenta, prior to exhibiting any observable fetal growth deficit [17, 25]. Given that ER stress and placental insufficiency were observed to precede human intrauterine growth restriction [26, 27, 29, 31, 33], the presence of augmented ER stress exhibited in e15 nicotine-exposed rat placentas reveal a potential mechanism through which nicotine may cause adverse placental and fetal outcomes in pregnant mothers who are smoking or undergoing nicotine replacement therapy.

An elegantly conducted study by DuRose et al. (2006) revealed intrinsic differences between the three UPR pathways (Atf6, IRE1α, and PERK) in their abilities to sense and recognize distinct types of ER perturbations [82]. We demonstrated that maternal nicotine exposure selectively activates the PERK unfolded protein response pathway in e15 rat placentas. Increases in the steady-state levels of Atf6 mRNA proposes possible involvement of the Atf6 branch, however, the lack of change in the protein levels and transcript levels of downstream target (*e*.*g*., Grp78), do not strongly support this at this particular time-point. Activation of PERK induces phosphorylation of eIF2α, which attenuates global protein translation to reduce the incoming protein load [83–85]. Phosphorylated eIF2α also paradoxically elevates translation of mRNA transcripts with conserved upstream open reading frames, such as Atf4, which was demonstrated through unchanged Atf4 mRNA levels, but significantly increased protein levels in our nicotine-treated placentas compared to controls [86–88]. Interestingly, Grp78 protein levels were also found to be significantly up-regulated in nicotine-treated placentas compared to controls, amidst unchanged mRNA levels. Grp78 mRNA transcription is more commonly regulated by Atf6 and IRE1α branches of the UPR; however, various post-transcriptional mechanisms (*e*.*g*., alternative translation initiation due to eIF2α phosphorylation) have been demonstrated to independently regulate protein levels of Grp78 in the presence of ER stress regardless of transcript levels [89, 90].

CHOP, which may be up-regulated by Atf4 and/or phosphorylated eIF2α, is known to amplify various downstream apoptosis pathways (*e*.*g*., down-regulation of anti-apoptotic Bcl-2 expression, translocation of Bcl-2-associated X protein (Bax) to mitochondria to amplify death pathway, etc.) [38, 71, 91–95]. However, the minimal changes seen in the ratio of Bax: Bcl-2 expression despite significantly increased CHOP expression perhaps suggests an early stage of CHOP activation in e15 nicotine-exposed placentas, when downstream apoptosis have not yet been fully elicited. The other possibility is that although CHOP is involved in the translocation of Bax to the mitochondria, it may not be involved in up-regulating the transcription of Bax [94]. The lack of change seen in other caspase markers further indicates the absence of nicotine effects on these specific apoptosis pathways at this particular time point. However, the expression of placental genes previously found to be influenced by nicotine (*e*.*g*., up-regulation of VEGF and down-regulation of Glut-1) are also altered in the same manner by tunicamycin (a known inducer of ER stress), without any reported activation of apoptosis [17, 29, 96, 97]. This collectively suggests that the structural and morphological aberrations in nicotine-exposed placentas may be due to the ER stress-induced alterations in gene expression caused by nicotine, instead of pathological apoptosis [17]. Therefore, the nicotine-induced unfolded protein response at e15 may possibly be attempting to avoid apoptosis by re-establishing some manner of sub-optimal placental homeostasis to adapt to the ER stress experienced.

Activation of the unfolded protein response also reveals possible dysfunction of protein maturation. Disulfide bond formation is critical for successful co- and post-translational modifications during protein maturation, and impairment is known to lead to ER stress [35, 98]. Traditionally, PDI and/or QSOX1 expression increases during hypoxia, tunicamycin or thapsigargin-induced ER stress to further assist with protein folding and disulfide bond formation [99–102]. PDI has also been found to be up-regulated in the lungs of chronic smokers, perhaps as a protective response against the oxidative damage of chronic cigarette smoke exposure [103–105]. However, down-regulation of mRNA transcripts of essential isomerases and oxidoreductases in disulfide bond formation (*e*.*g*., VKORC1, QSOX1) was seen in the nicotine-exposed rat placentas at e15. Protein levels were also seen to be significantly down-regulated in a few markers (*e*.*g*., PDI, QSOX1), though to a lesser degree compared to the change in mRNA levels. It is possible that these transcripts initially down-regulated by nicotine are being subsequently stabilized at the protein level by the unfolded protein response, which seeks to post-transcriptionally up-regulate their protein levels in the presence of ER stress, as seen in previous studies [99–105]. This may explain the milder changes seen in protein levels of VKORC1 and QSOX1, despite strong decreases in their transcript levels. Interestingly, cigarette smoke has recently been demonstrated to also lead to excessive posttranslational oxidation of PDI, abating its functionality in the formation of disulfide bonds [60]. Given that inhibition of PDI is also known to disrupt protein folding and augment ER stress, it may be stipulated that the nicotine-induced down-regulation of PDI and other oxidoreductases at e15 may still be contributing in part to the augmentation of ER stress, despite the adaptive efforts of the unfolded protein response to stabilize their protein levels [46]. Regardless, additional studies must first be conducted on the individual effects of nicotine and UPR activation on PDI and oxidoreductase regulation to further address these speculations.

It is noteworthy that increased expression of Hif1α, alongside previously reported increases in CA-IX expression, jointly reveals hypoxia in nicotine-exposed placentas [17, 78, 79]. The increase in hypoxia may be due to nicotinic antagonism of nAChR α9, which induces vasoconstriction of placental vasculature to reduce oxygen supply [48, 49]. Koritzinsky et al. (2013) recently identified oxygen as the terminal electron acceptor in post-translational disulfide bond formation, further implicating the impairment of protein maturation in hypoxia-induced ER stress [52]. Additionally, vasoconstriction is known to reduce nutrient and amino acid supply to the placenta [48]. Nicotine has also been documented to depress amino acid transport system A and block acetylcholine-mediated nutrient delivery in trophoblasts, collectively hindering the maternal transport of many essential amino acids to the feto-placental unit [106–108]. Significantly increased expression of GCN2 indeed reveals amino acid starvation in nicotine-exposed placentas [80, 81]. Furthermore, GCN2 is an alternative kinase of eIF2α and may be partially responsible for its phosphorylation alongside PERK to cooperatively initiate an integrated stress response to hypoxia/ER stress and amino acid starvation [109]. However, future research is directed to further investigate the relationships between low amino acid supply and impaired disulfide bond formation.

**Fig 8:**
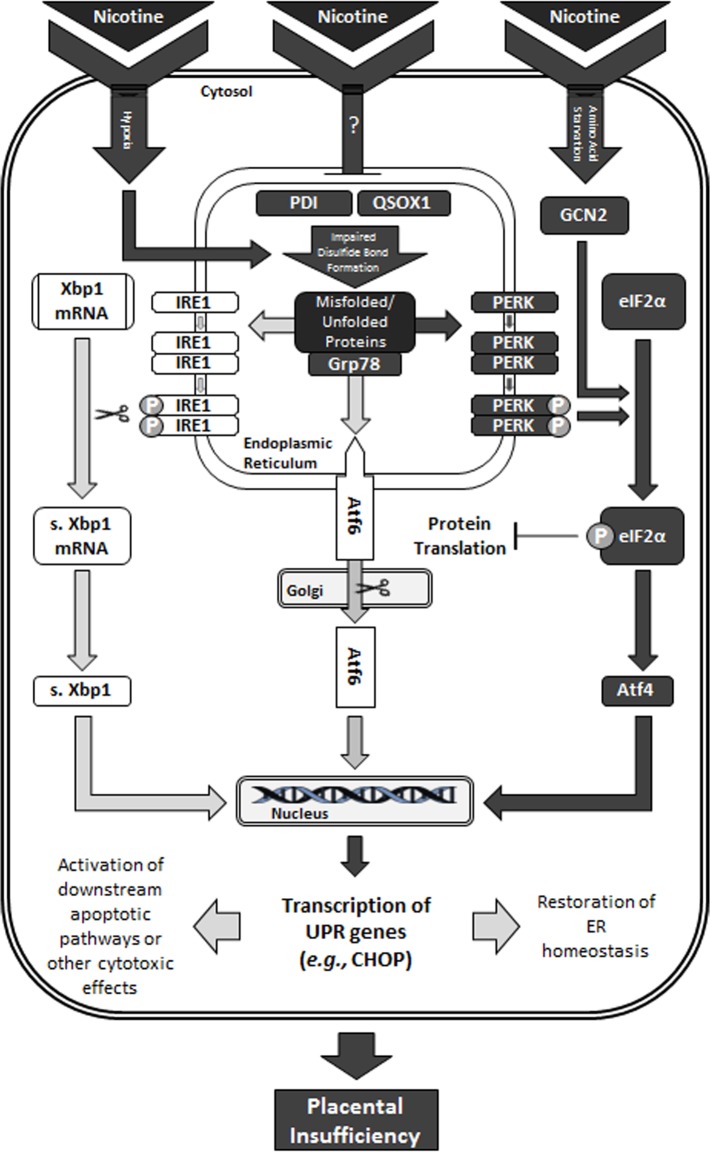
Proposed schematic of the effect of nicotine on ER stress and the unfolded protein response in the e15 placenta. Pathways affected by nicotine are indicated by the darkened arrows and boxes. In summary, nicotine exposure was shown to augment ER stress and activate the unfolded protein response in the e15 placenta. Activation was most prominent in the PERK branch and was demonstrated in association with impaired disulfide bond formation. Nicotine is proposed to impair disulfide bond formation through direct or indirect down-regulation of PDI and other oxidoreductases. Disulfide bond formation is further impaired through increased hypoxia as caused by nicotine-induced vasoconstriction. Additionally, up-regulation of GCN2 suggests amino acid starvation and activation of the integrated stress response to further phosphorylate eIF2α. However, the lack of Bax and caspase activation seen at e15 suggests that the nicotine-induced ER stress response may possibly be attempting to avoid apoptosis by re-establishing some manner of sub-optimal placental homeostasis to adapt to the ER stress experienced.

In summary, this study has demonstrated that nicotine alone can induce ER stress and evoke an integrated stress response in the rat placenta, as revealed through PERK- and GCN2-activation of the p-eIF2α-Atf4-CHOP axis (Fig. 8). Recent studies have demonstrated the induction of ER stress via super-physiological nicotine dosages or cigarette smoke [55–61]; however, our study was the first to induce ER stress in the rat placenta through an *in vivo* model of maternal nicotine exposure using physiological nicotine dosages. Furthermore, we provide novel insight by demonstrating this in association with impairment of the disulfide bond formation pathway, as shown through nicotine-induced down-regulation of PDI and QSOX1 expression and increased hypoxia. By elucidating that maternal nicotine exposure is linked to placental ER stress and impaired disulfide bond formation, this may contribute to the development of more efficacious interventions (*e*.*g*., Tauroursodeoxycholic acid to relieve ER stress [110]). More importantly, given that nicotine alone exerts severe effects on placental function, and consequently, on fetal and postnatal health, this study further implicates that greater caution is required for women considering nicotine replacement therapy for smoking cessation in pregnancy.

## Supporting Information

S1 Fig mRNA targets all normalized to individual housekeeping genes selected for geometric mean. Trends remain across all normalizations to individual housekeeping genes. All mRNA levels were expressed as means normalized to either β-Actin, 18S, Gapdh, or the geometric mean ± SEM (n = 5-6/group). Statistical analyses were not performed in these graphs.
(TIF)
