# Supplementary figures and images for: Maternal Nicotine Exposure Leads to Impaired Disulfide Bond Formation and Augmented Endoplasmic Reticulum Stress in the Rat Placenta
Source: PLoS One. 2015 Mar 26;10(3):e0122295. doi: 10.1371/journal.pone.0122295 (PMC4374683; doi:10.1371/journal.pone.0122295)

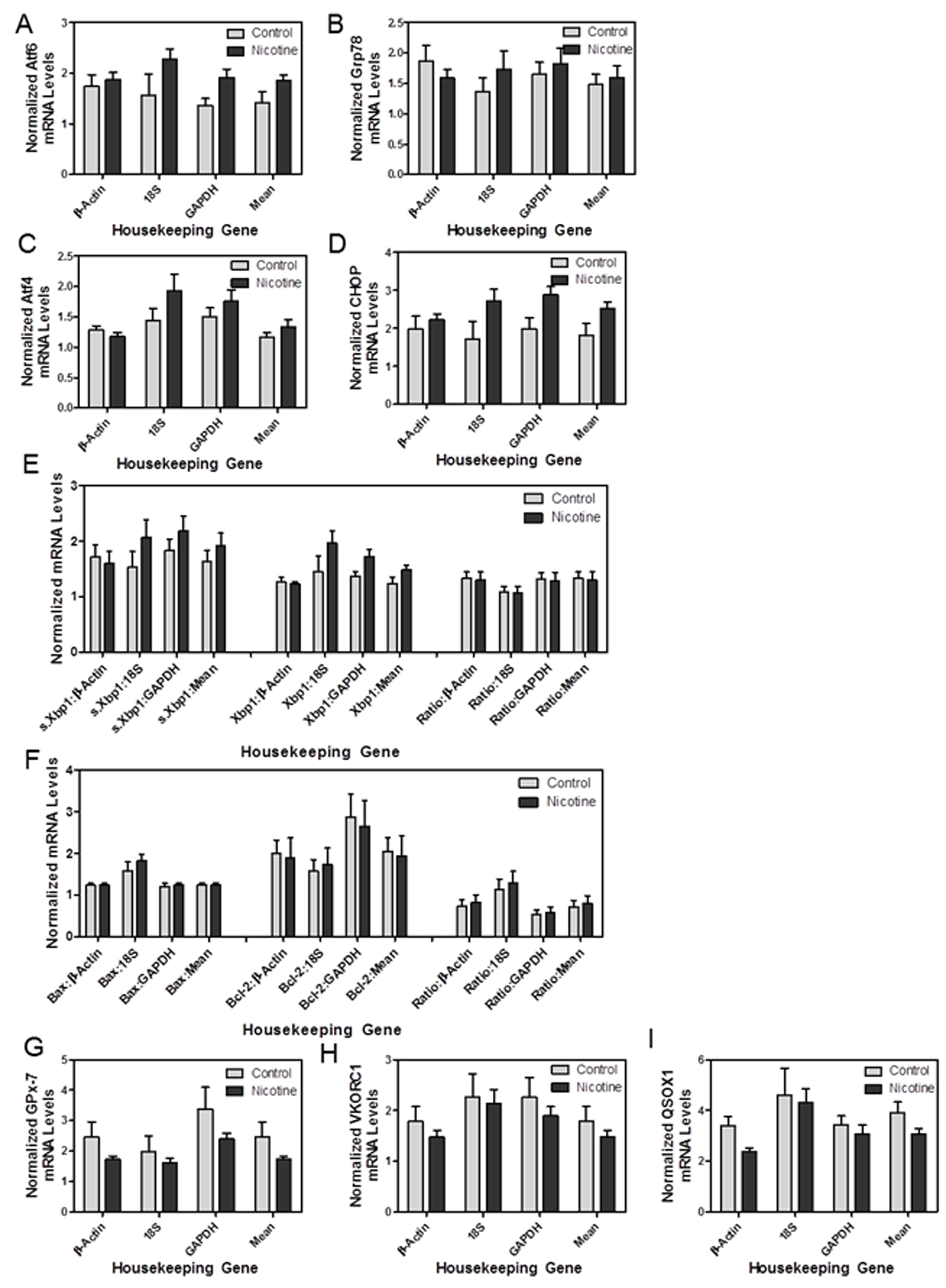

Supplement: S1 Fig — Trends remain across all normalizations to individual housekeeping genes. All mRNA levels were expressed as means normalized to either β-Actin, 18S, Gapdh, or the geometric mean ± SEM (n = 5-6/group). Statistical analyses were not performed in these graphs. (TIF) [file pone.0122295.s001.tif]
